# Novel Descattering Approach for Stereo Vision in Dense Suspended Scatterer Environments

**DOI:** 10.3390/s17061425

**Published:** 2017-06-17

**Authors:** Chanh D. Tr. Nguyen, Jihyuk Park, Kyeong-Yong Cho, Kyung-Soo Kim, Soohyun Kim

**Affiliations:** 1 Department of Mechanical Engineering, KAIST, 291 Daehak-ro, Yuseong-gu, Daejeon 34141, Korea; chanhnguyen@kaist.ac.kr (C.D.T.N.); jihpark@kaist.ac.kr (J.P.); 2 Unmanned Safety Robot Research Center, KAIST, 291 Daehak-ro, Yuseong-gu, Daejeon 34141, Korea; yong00@kaist.ac.kr

**Keywords:** low visibility, backscattering, descattering, defogging, stereo vision

## Abstract

In this paper, we propose a model-based scattering removal method for stereo vision for robot manipulation in indoor scattering media where the commonly used ranging sensors are unable to work. Stereo vision is an inherently ill-posed and challenging problem. It is even more difficult in the case of images of dense fog or dense steam scenes illuminated by active light sources. Images taken in such environments suffer attenuation of object radiance and scattering of the active light sources. To solve this problem, we first derive the imaging model for images taken in a dense scattering medium with a single active illumination close to the cameras. Based on this physical model, the non-uniform backscattering signal is efficiently removed. The descattered images are then utilized as the input images of stereo vision. The performance of the method is evaluated based on the quality of the depth map from stereo vision. We also demonstrate the effectiveness of the proposed method by carrying out the real robot manipulation task.

## 1. Introduction

High spatial resolution ranging is crucial in robot manipulation and a depth map is necessary to accomplish the task. There are many cases where the system works in low visibility and strong scattering environments, such as underwater robots or firefighting robots. Our application is bipedal and quadrupedal robots working in nuclear power plants where they must cope with poor visibility due to dense steam. When an accident occurs, the plant is filled with very dense water-based atmospheric particles, and the robot needs to operate the plant. From our experiments, the commonly used sensors such as LiDAR (LMS511, SICK, Waldkirch, Germany and UTM-30LX-EW, Hokuyo, Osaka, Japan) and time of flight (ToF) camera (Kinect v2, Microsoft, Redmond, WA, USA) are unable to work in such low visibility conditions. Our conclusion is consistent with the study by Starr and Lattimer [1]. Some specialized subsea LiDAR systems (please refer to Massot-Campos and Oliver-Codina [2] for a comprehensive survey of underwater 3D reconstruction), laser line scanning [3] or structured light [4,5,6,7] are able to operate in scattering media. However, these systems are power-consuming, slow, and bulky and thus they are not well suited for a walking robot. Our goal is to utilize the images from a standard stereo vision system for robot manipulation in a scattering environment. Therefore, no additional hardware is required for the stereo vision system.

Stereo vision has been intensively studied for decades since retrieving the depth map of a scene is critical in many applications such as driving assistance and automated robotics. However, most state-of-the-art methods of stereo vision primarily deal with high-quality images from datasets, for example, Middlebury datasets [8,9], and focus on either reducing the matching error or providing a real-time system [8,10,11]. Most stereo vision algorithms follow the multi-stage framework codified by Scharstein and Szeliski [8]. The rectified images should pass four main sequential steps to obtain the disparity map. The four stages are matching cost computation, cost aggregation, disparity selection, and disparity refinement. In general, the stereo algorithms can be classified into two categories, namely, local and global approaches. In a local approach, the disparity computation at given point depends only on the intensity value within the local window of grayscale images [12] or color images [13]. It has low computational complexity and short running time. These methods commonly have an inherent conceptual problem that it is assumed that the region inside the window is a fronto-parallel surface and does not cover the depth discontinuities. Studies based on varying support-weights window [14] or the geodesic support weights [15] can overcome the problem of depth discontinuities but are time-consuming. A more recent approach [16] that utilized guided filtering [17] achieved start-of-the-art results very efficiently. In a global approach, the problem, on the other hand, is formulated as a global optimization problem. In this approach, second and third steps are combined, and the main difference lies in how the optimization problem is solved. The problem can be solved efficiently using graph cut [18,19] and loopy belief propagation [20,21], among others. However, these methods, in practice, are rather slow. Both local and global methods use the photo-consistency constraint to find the corresponding pixels. In other words, they try to find the most similar pixel intensities in the left and right image.

These methods, however, cannot be applied directly to images taken in an indoor dense scattering environment where active light sources are required for illumination. The reason is that the scene radiance is attenuated while it propagates before reaching the camera. The greater the distance is, the weaker the signal that the camera receives is. Therefore, the image contrast is low. Additionally, the cameras capture the scattering signal, which increases with the object distance, scattered by the suspended particles. Furthermore, using non-parallel and non-uniform artificial illumination sources, situated close to the cameras, generates a significant backscattering signal, which is spatially-varying, under a geometric constraint. Thus, the intensities of the same object captured by two cameras of the system can be significantly different. Therefore, the photo-consistency does not hold. For the close range measurement in our case, stereo vision wrong matching is mainly due to backscattering rather than low contrast. Thus, non-uniformity of the backscattering is the dominant cause of wrong matching in stereo vision.

The stereo vision of a natural foggy scene can take advantage of developed image visibility enhancement methods. The polarization-based method enhanced the haze images under natural light [22] or underwater images utilizing active polarized light [23,24] by examining the degree of polarization (DOP) from multiple images taken under different polarization states. The methods are based on the assumption that the DOP of the object is spatial-invariant, which does not hold in our case. There has been significant progress in single image removal of haze, a process called dehazing, based on Koschmieder’s law [25]. Markov random field (MRF) was used as a framework to derive the cost function by Tan [26], Fattal [27], and Nishino et al. [28]. Based on natural image statistics, the well-known Dark Channel Prior (DCP) was derived by He et al. [29]. Owing to DCP’s effectiveness in dehazing, the majority of start-of-the-art dehazing techniques [30,31,32] have adopted the prior. Recently, learning-based methods [33,34] have also been utilized to solve image dehazing problems, providing state-of-the-art results. These methods, except for [23,24], targeted corrupted images primarily caused by attenuation rather than non-uniform backscattering from active illumination.

Recently, several nighttime dehazing algorithms have been developed. Zhang et al. [35] utilize a new imaging model to compensate light and correct color before applying DCP. Li et al. [36] incorporate a glow term into standard nighttime haze model. After the glow is decomposed from the image, DCP is employed to obtain a haze-free image. These methods can be utilized in a pre-processing step for stereo vision in a scattering scene with active light sources. However, they are not real-time capable.

Several methods were introduced to solve stereo vision for images of fog or underwater scenes. Caraffa and Tarel [37] combine photo-consistency term and atmosphere veil depth cues to formulate the problem and solve stereo and defog by utilizing the α-expansion algorithm [18]. This method is sensitive to the nonlinear camera respond function and image noise. Therefore, the authors demonstrated proper results for synthetic images but not real foggy images. Roser et al. [38] iterate applying a conventional stereo algorithm to compute the depth and using depth to recover the object radiance. The method, however, does not model light scattering in the stereo matching step and defogs video frames independently, which cause errors in stereo matching. Li et al. [39] solve depth reconstruction and defog simultaneously from monocular video based on structure-from-motion (SfM). This only works when SfM can be calculated. Furthermore, the method is far from real-time capable since 10 min per frame is reported. These studies noted above are capable of processing images obtained under natural light sources only. Negahdaripour and Sarafraz in [40] use both photo-consistency and backscattering cues to estimate disparity by the local matching method. The method can be applied to images corrupted by backscattering, taken under a non-homogeneous artificial light source. The authors, however, assumed that the depth in the supported window is constant, which led to wrong estimation of the scattering signal at depth discontinuities, especially in a highly non-homogeneous scattering signal area. 

In this study, we propose a scattering removal technique, called *descattering*, followed by a standard stereo method where we focus on how to remove the scattering efficiently for stereo vision. The imaging model is derived in Section 2. From the model, the model-based descattering method is proposed, where we try to remove the scattering effect. The intermediate resulting images of the descattering method are defogged utilizing the well-known DCP [29]. Both steps above are shown in Section 3. The results of stereo vision of dense scattering scene of both synthetic images and real experimental images are shown in Section 4. The robot system and the accomplishment of a robot manipulation task are demonstrated in Section 5. Finally, Section 6 presents our conclusions.

## 2. Imaging Model

Three underlying assumptions are used in this approach:

- The illumination source is known and close to the cameras. This is feasible since the cameras and the light source are installed in the head of the robot.
- The scattering is single scattering. Although multiple scattering occurs, it is proven that utilizing single scattering model is effective in scattering removal [24,40,41].
- The input image **I** is given in the actual scene radiance values. The radiance maps can be recovered by inverting the acquisition response curve proposed by Debevec and Malik [42].

### 2.1. Single View Modeling in Scatterers Environment

**Figure 1:**
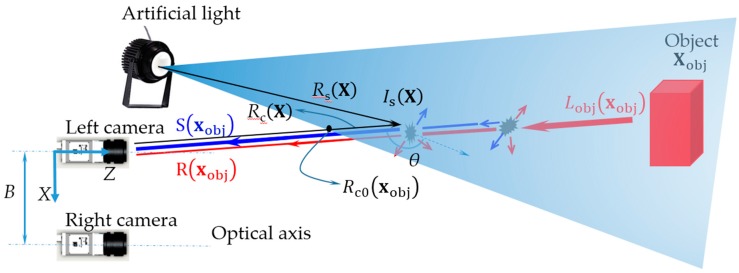
Stereo vision system configuration.

Consider a vision system configuration in Figure 1. Let X=(X,Y,Z) and x=(x,y) be global coordinates of a point in space and its projection into image plan, respectively. $R_{s}\left(X\right)$ and $R_{c}\left(X\right)$ are the distances from a point in space X to the light source and the left camera, respectively. $R_{c0}\left(x_{\mathrm{obj}}\right)$ is the distance where the light field first intersects the line of sight (LOS), which is unique for every pixel $x_{\mathrm{obj}}$ in the image. $I_{s}\left(X\right)$ is the irradiance of a point in space that illuminated by the point light source and θ is the backscattering angle. *B* is the baseline. The measured intensity can be modeled as a linear combination of attenuated radiance $R\left(x_{\mathrm{obj}}\right)$ (red line) (attenuated fraction of object radiance $L_{\mathrm{obj}}\left(x_{\mathrm{obj}}\right)$) and backscattering component $S\left(x_{\mathrm{obj}}\right)$ (blue line) as follows:

(1) $$I\left(x_{\mathrm{obj}}\right)=\;R\left(x_{\mathrm{obj}}\right)+S\left(x_{\mathrm{obj}}\right).$$

Note that the single scattering is assumed and the image blur due to the forward scattering [41] is not taken into account. The attenuated signal is:

(2) $$R\left(x_{\mathrm{obj}}\right)=L_{\mathrm{obj}}\left(x_{\mathrm{obj}}\right)\tau \left(x_{\mathrm{obj}}\right)$$

where direct transmission is:

(3) $$\tau \left(x_{\mathrm{obj}}\right)=e^{-cR_{c}\left(X_{\mathrm{obj}}\right)}$$

where c is the attenuation coefficient (or extinction coefficient) of the environment due to absorption and scattering. The object radiance is given by:

(4) $$L_{\mathrm{obj}}\left(x_{\mathrm{obj}}\right)=I_{s}\left(X_{\mathrm{obj}}\right)\rho \left(X_{\mathrm{obj}}\right)$$

where $\rho \left(x_{\mathrm{obj}}\right)$ is the object reflectance. The irradiance of a point in space that illuminated by the point light source of intensity $L_{s}$ is:

(5) $$I_{s}\left(X\right)=\frac{L_{s}Q\left(X\right)}{R_{s}^{2}\left(X\right)}e^{-cR_{s}\left(X\right)}$$

where Q(X) expresses the non-uniformity of the illumination source. The falloff 1Rs2(X) is caused by free space light propagation. Since the baseline of the illuminator-camera is very small compared to the object distance, $R_{s}\left(X\right)\approx R_{c}\left(X\right)=\|X\|$. Substituting Equations (3)–(5) into Equation (2), we obtain:

(6) $$R\left(x_{\mathrm{obj}}\right)=\frac{L_{s}Q\left(X_{\mathrm{obj}}\right)}{{\left\|X_{\mathrm{obj}}\right\|}^{2}}\rho \left(X_{\mathrm{obj}}\right)e^{-2c\|X_{\mathrm{obj}}\|}.$$

The total backscattering signal that the camera receives is:

(7) $$S\left(x_{\mathrm{obj}}\right)=\int_{R_{c0}\left(x_{\mathrm{obj}}\right)}^{R_{c}\left(X_{\mathrm{obj}}\right)}b\left[\theta \left(X\right)\right]I_{s}\left(X\right)e^{-cR_{c}\left(X\right)}dR_{c}\left(X\right),\;X\in X_{\mathrm{LOS}}$$

where b[θ(X)] is the phase function of backscattering. The LOS from the camera to object is:

(8) $$X_{\mathrm{LOS}}=\left\{X:X=x_{\mathrm{obj}}\frac{Z}{f},Y=y_{\mathrm{obj}}\frac{Z}{f},Z\in \left[0,Z_{\mathrm{obj}}\right]\right\}$$

where *f* is the cameras’ focal length. To simplify the analysis, let us assume that $b\left[\theta \left(X_{\mathrm{LOS}}\right)\right]\approx \tilde{b}$ is constant over the field of view, which is supported by [23,24], and that $Q\left(X\right)\approx Q\left(X_{\mathrm{obj}}\right)$ is constant along the LOS, which is supported by small camera-illuminator baselines. If there are several sources, Equation (7) applies to each source. Accumulating the integral for all sources yields the total backscattering. Equation (7) becomes:

(9) $$S\left(x_{\mathrm{obj}}\right)=\;\tilde{b}L_{s}Q\left(X_{\mathrm{obj}}\right)\int_{R_{c0}\left(x_{\mathrm{obj}}\right)}^{R_{c}\left(X_{\mathrm{obj}}\right)}\frac{e^{-2cR_{c}\left(X\right)}}{R_{c}^{2}\left(X\right)}dR_{c}\left(X\right),\;X\in X_{\mathrm{LOS}}.$$

Tribitz and Schechner [23] derived the analytic solution of the integral in Equation (9) as Equation (10) and its approximation as Equation (11):

(10) $$\frac{S\left(x_{\mathrm{obj}}\right)}{\tilde{b}L_{s}Q\left(X_{\mathrm{obj}}\right)}\approx {\left\{-\frac{e^{-2cR_{c}\left(X\right)}}{R_{c}\left(X\right)}-2c\ln R_{c}\left(X\right)-2c\overset{\infty }{\underset{n=1}{\sum }}\frac{{\left[-2cR_{c}\left(X\right)\right]}^{n}}{n.n!}\right\}|}_{R_{c}\left(X\right)=R_{c0}\left(x_{\mathrm{obj}}\right)}^{R_{c}\left(X\right)=R_{c}\left(X_{\mathrm{obj}}\right)},\;X\in X_{\mathrm{LOS}},$$

(11) $$S\left(x_{\mathrm{obj}}\right)\approx S_{\infty }\left(x_{\mathrm{obj}}\right)\left\{1-e^{-k\left[R_{c}\left(X_{\mathrm{obj}}\right)-R_{c0}\left(x_{\mathrm{obj}}\right)\right]}\right\}$$

where $S_{\infty }\left(x_{\mathrm{obj}}\right)\propto \tilde{b}L_{s}Q\left(X_{\mathrm{obj}}\right)$ (considering Equation (10)) denotes the saturated backscattering value. It is worth noting that the non-uniformity of $S_{\infty }\left(x_{\mathrm{obj}}\right)$ is attributed to the anisotropic pattern $Q\left(X_{\mathrm{obj}}\right)$ in this special case. The constant parameter *k* depends on $R_{c0}\left(x_{\mathrm{obj}}\right),$ *c* and $\tilde{b}$. From Equation (11), the rate at which $S\left(x_{\mathrm{obj}}^{i}\right)$ increases with $\left\|X_{\mathrm{obj}}^{i}\right\|$ is set by parameter *k*. Since the baseline of illuminator-camera is very small compared to the object distance, in widefield lighting, we have $R_{c0}\left(x_{\mathrm{obj}}\right)\ll R_{c}\left(X_{\mathrm{obj}}\right)$, thus $R_{c0}\left(x_{\mathrm{obj}}\right)\approx 0$. Substituting Equations (6) and (11) into Equation (1), noting that $R_{c}\left(X_{\mathrm{obj}}\right)=\|X_{\mathrm{obj}}\|$, the image’s intensity becomes:

(12) $$I\left(x_{\mathrm{obj}}\right)=\;\frac{L_{s}Q\left(X_{\mathrm{obj}}\right)}{{\left\|X_{\mathrm{obj}}\right\|}^{2}}\rho \left(X_{\mathrm{obj}}\right)e^{-2c\|X_{\mathrm{obj}}\|}+S_{\infty }\left(x_{\mathrm{obj}}\right)\left(1-e^{-k\|X_{\mathrm{obj}}\|}\right).$$

Equation (12) resembles Koschmieder’s law, which models daytime outdoor fog. The major difference is that in our case $S_{\infty }\left(x_{\mathrm{obj}}\right)$ is spatial variant.

### 2.2. Stereo Modeling in Suspended Scatterer Environment

In a stereo vision system, using Equations (4) and (5), Equation (12) becomes:

(13) $$I^{i}\left(x_{\mathrm{obj}}^{i}\right)=L_{\mathrm{obj}}^{i}\left(x_{\mathrm{obj}}\right)e^{-c\|X_{\mathrm{obj}}^{i}\|}+S_{\infty }^{i}\left(x_{\mathrm{obj}}^{i}\right)\left(1-e^{-k\|X_{\mathrm{obj}}^{i}\|}\right)$$

where $X_{\mathrm{obj}}^{i},\;i=L,\;R$ are the coordinates of a point $X_{\mathrm{obj}}$ in space with respect to left and right cameras, respectively. Noting that the global coordinates and left camera coordinates are the same ($X_{\mathrm{obj}}^{L}=X_{\mathrm{obj}}$), we have the relationship:

(14) $$X_{\mathrm{obj}}^{R}=X_{\mathrm{obj}}^{L}-\left(B,0,0\right).$$

In Equation (13), the image coordinates of a point in space projected into the rectified left and right images are $x_{\mathrm{obj}}^{i}$, *i* = L, R. We also have:

(15) $$x_{\mathrm{obj}}^{R}=x_{\mathrm{obj}}^{L}-\left[d\left(x_{\mathrm{obj}}^{L}\right),0\right]$$

where $d\left(x_{\mathrm{obj}}^{L}\right)$ is the disparity map that pairs up corresponding pixels $\left\{x_{\mathrm{obj}}^{L},x_{\mathrm{obj}}^{R}\right\}$. 

In general, the system setup is more complicated than what was derived in Section 2.1: lighting geometry cannot be ignored, and there are several sources. In such cases, Tribitz and Schechner [23,24] show that the backscatter still follows the approximated model in Equation (11). However, $S_{\infty }^{i}\left(x_{\mathrm{obj}}^{i}\right)$ depends not only on the anisotropic pattern $Q\left(X_{\mathrm{obj}}^{i}\right)$ of the light source and the scattering parameters *c* and $\tilde{b}$, but also on the lighting geometry. The smaller camera-illuminator baseline is, the stronger the non-uniformity is. Equation (5) shows that the LOS that is closer to the light source receives stronger backscattering signal. The reason is that the irradiance $I_{s}\left(X\right)$ is very strong where the light field first meet the LOS. Thus, the two cameras sense a different backscattering signal, depending on their geometric relationships to the light. That makes the stereo vision in scattering media more problematic because the intensity of the same object can be significantly different. 

**Figure 2:**
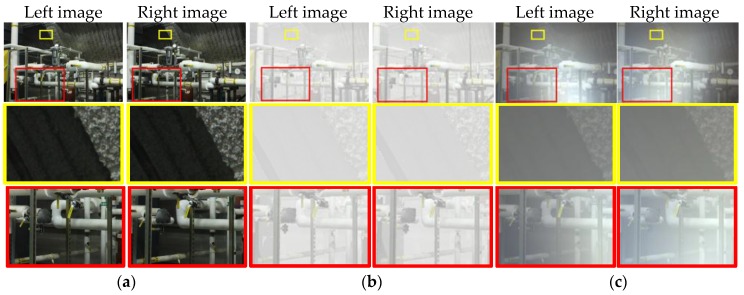
Example of stereo images (pipes from the Middlebury 2014 stereo datasets [9]) in different environment: (**a**) Stereo pair taken in clean environment; (**b**) Stereo pair taken in foggy environment with natural light source that suffers from attenuation uniform scattering; (**c**) Stereo pair taken in dense scatterer environment under an active light source that suffers both attenuation and non-uniform backscattering.

Figure 2a–c show the stereo pair of a clear scene, a foggy scene with natural light, and a foggy scene with an artificial light source, respectively. In the first example, since the images are taken in a clean environment, texture and contrast are preserved. Therefore, these images can be directly processed by utilizing conventional well-developed stereo vision algorithms. Figure 2b depicts the synthetic stereo images of the scene shown in Figure 2a in the presence of fog under natural light. In this case, the imaging model obeys Koschmieder’s law [25]. Due to attenuation, the greater the distance the signal propagates over, the weaker the object radiance that the cameras receive is. Thus, the contrast of these objects (inside the yellow rectangle) is low. Additionally, since the natural light is assumed to be parallel and uniform, the cameras capture the scattering signal, which depends on the air attenuation coefficient and the object distance. Although there are some difficulties in obtaining a depth map from these images due to poor contrast, the photo-consistency is held. Figure 2c represents an even more complicated case: the synthetic images of a scene under a foggy condition illuminated by an artificial light source that is installed under the two cameras. Besides suffering from poor contrast due to attenuation, the light adds a different scattering signal to the cameras, depending on lighting geometry. Consequently, the brightness of one object in two images (inside red rectangle) is not identical. Thus, the photo-consistency does not hold.

## 3. Backscattering and Fog Removal

### 3.1. Non-Uniform Backscattering Removal

#### 3.1.1. Light Compensation

The first step is light compensation, which removes the non-uniformity of the backscattering. To do this, the measured image, modeled in Equation (13), is divided by the saturated backscattering signal, we obtain a light compensated image:

(16) $${\hat{J}}^{i}\left(x_{\mathrm{obj}}^{i}\right)=\;{\hat{L}}_{\mathrm{obj}}^{d,i}\left(x_{\mathrm{obj}}^{i}\right)e^{-k\|X_{\mathrm{obj}}^{i}\|}+\left(1-e^{-k\|X_{\mathrm{obj}}^{i}\|}\right)$$

where the distorted radiance of object is defined as:

(17) $${\hat{L}}_{\mathrm{obj}}^{d,i}\left(x_{\mathrm{obj}}^{i}\right)=\frac{L_{\mathrm{obj}}^{i}\left(x_{\mathrm{obj}}\right)}{S_{\infty }^{i}\left(x_{\mathrm{obj}}^{i}\right)}e^{-\left(c-k\right)\|X_{\mathrm{obj}}^{i}\|}$$

where $L_{\mathrm{obj}}^{i}\left(x_{\mathrm{obj}}\right)/S_{\infty }^{i}\left(x_{\mathrm{obj}}^{i}\right)$ is a spatial-varying value. It depends on the geometric configuration of the light. This means that applying the light compensation step results in many local radiometric differences in the object signal. However, it will be compensated after defogging. The light compensated image in Equation (16) is similar to Koschmieder’s law with the airlight equal 1. Let us denote $\tau^{k,i}\left(x_{\mathrm{obj}}^{i}\right)=e^{-k\|X_{\mathrm{obj}}^{i}\|}$ , which is the modified direct transmission.

Noting that ${\hat{L}}_{\mathrm{obj}}^{d,i}\left(x_{\mathrm{obj}}^{i}\right)$ in Equation (17) is neither the reflectivity nor radiance of the object. It, however, is an enhanced image, with radiometric distortion, from the original corrupted image by strong backscattering. 

#### 3.1.2. Saturated Backscattering Estimation

Based on (11), saturated backscattering can be easily estimated:

(18) $$\underset{\|X_{\mathrm{obj}}^{i}\|\to \infty }{\lim}I^{i}\left(x_{\mathrm{obj}}^{i}\right)=S_{\infty }^{i}\left(x_{\mathrm{obj}}^{i}\right).$$

Thus, saturated backscattering can be pre-calibrated by taking the void images where there is no object in the images $\left(\|X_{\mathrm{obj}}^{i}\|\to \infty \right)$.

However, in our experiment, due to space limitation, we took pictures of very dense steam and fog (e.g., $c\approx 2.3{\;m}^{-1}$) scenes where no object can be seen. As derived in Section 2.2, saturated backscattering depends on the attenuation coefficient c. However, from Equation (10), we can obtain the following relationship:

(19) $$S_{\infty }^{i}\left(x_{\mathrm{obj}}^{i},c<2.3\right)\approx k_{c}S_{\infty }^{i}\left(x_{\mathrm{obj}}^{i},c=2.3\right)$$

where $k_{c}<1$ is the constant gain, which depends on the attenuation coefficient. The constancy of $k_{c}$ at the specific attenuation coefficient was confirmed by our experiment, for example, $k_{c=1.5}=0.86\pm 0.05$. 

**Figure 3:**
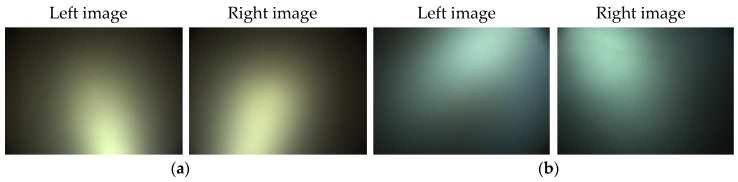
An example of saturated backscattering: (**a**) Lighting setup 1; (**b**) Lighting setup 2.

Figure 3 illustrates the saturated backscattering signal of two different system configurations. The images are the original images without any color correction. In the first setup, the light was put under the cameras, and steam was generated by the steam generator using pure water. The light was placed above the cameras in the second setup, and the fog was produced by a fog machine using oil.

### 3.2. Defogging

#### 3.2.1. DCP-Based Defogging

DCP [29] is employed to remove the fog, a process called defogging, of the light compensated image in Equation (16). Let us summarize the DCP proposed by He et al. [29]. The dark channel of the light compensated image is defined as:

(20) $${\tilde{J}}^{d}\left(x_{0}\right)=\underset{x\epsilon Ω\left(x_{0}\right)}{\min}\left[\underset{c\epsilon \left\{r,g,b\right\}}{\min}{\hat{J}}^{c}\left(x\right)\right]$$

where $Ω\left(x_{0}\right)$ is the local patch centered at $x_{0}$. The patch transmission is then calculated as:

(21) $${\tilde{\tau }}^{k}\left(x\right)=1-{\tilde{J}}^{d}\left(x\right).$$

Different from the original DCP method, we employ guided image filtering [17] to refine the raw transmission map in Equation (21) in order to obtain $\tau^{k}\left(x\right)$. The distorted object radiance can be obtained by inverting Equation (16):

(22) $${\hat{L}}_{\mathrm{obj}}^{d,i}\left(x_{\mathrm{obj}}^{i}\right)=\frac{\hat{J}\left(x_{\mathrm{obj}}^{i}\right)-1}{\max\left[\tau^{k,i}\left(x_{\mathrm{obj}}^{i}\right),\tau_{0}\right]}+1.$$

Transmission can be very close to zero; thus, it is restricted to the lower bound $\tau_{0}$. There is radiometric distortion in the distorted object radiance, as shown in Equation (17). Therefore, to preserve the photo-consistency in the left and right images, the radiometric distortion must be eliminated. This can be done easily by multiplying the distorted object radiance by saturated backscattering $S_{\infty }^{i}\left(x_{\mathrm{obj}}^{i}\right)$ to obtain the modified object radiance as follows:

(23) $${\hat{L}}_{\mathrm{obj}}^{i}\left(x_{\mathrm{obj}}^{i}\right)=L_{\mathrm{obj}}^{i}\left(x_{\mathrm{obj}}\right)e^{-\left(c-k\right)\|X_{\mathrm{obj}}^{i}\|}.$$

It is also worth noting that ${\hat{L}}_{\mathrm{obj}}^{i}\left(x_{\mathrm{obj}}^{i}\right)$ is not the original radiance of the object. The value $e^{-\left(c-k\right)\|X_{\mathrm{obj}}^{i}\|}$ either attenuates (when −(c−k)<0) or amplifies (when −(c−k)>0) the original object radiance. However, in our experiment, the modified radiance images are useful for both reviewing the scene and reconstructing depth map. For simplicity, we call ${\hat{L}}_{\mathrm{obj}}^{i}\left(x_{\mathrm{obj}}^{i}\right)$ a *defogged image* in our paper.

DCP was designed for natural images. The assumption may not hold for indoor human-made scenes. The main reason is that DCP can detect the specular reflection [43]. By utilizing the active polarization system [24] (explained in Appendix A), the specular reflection can be removed; thus, we verify that the DCP works properly in our system. 

#### 3.2.2. Normalization-Based Image Correction

From our observation, when the fog is very dense and uniform, the modified direct transmission $\tau^{k}\left(x_{\mathrm{obj}}\right)$ is almost a constant, which is very small; thus, the backscatter $S^{i}\left(x_{\mathrm{obj}}\right)$ is close to its saturation $S_{\infty }^{i}\left(x_{\mathrm{obj}}\right)$. Consequently, the minimum intensity of the light compensated image is set by atmospheric veil $\left[1-\tau^{k}\left(x_{\mathrm{obj}}\right)\right]$. Therefore, by normalizing the light compensated image ${\hat{J}}^{i}\left(x_{\mathrm{obj}}^{i}\right)$, we can efficiently both remove the atmospheric veil and scale ${\hat{L}}_{\mathrm{obj}}^{d,i}\left(x_{\mathrm{obj}}^{i}\right)$ to [0,1]. The normalization image is defined as follows:

(24) $${\hat{J}}_{n}^{i}\left(x_{\mathrm{obj}}^{i}\right)=\frac{{\hat{J}}^{i}\left(x_{\mathrm{obj}}^{i}\right)-\min\left[{\hat{J}}^{i}\left(x_{\mathrm{obj}}^{i}\right)\right]}{\max\left[{\hat{J}}^{i}\left(x_{\mathrm{obj}}^{i}\right)\right]-\min\left[{\hat{J}}^{i}\left(x_{\mathrm{obj}}^{i}\right)\right]}.$$

The image is an approximation of ${\hat{L}}_{\mathrm{obj}}^{d,i}\left(x_{\mathrm{obj}}^{i}\right)e^{-k\|X_{\mathrm{obj}}^{i}\|}$ in Equation (16). Then, to remove radiometric distortion, we define the compensated normalization image as:

(25) $${\hat{R}}_{n}^{i}\left(x_{\mathrm{obj}}^{i}\right)={\hat{J}}_{n}^{i}\left(x_{\mathrm{obj}}^{i}\right)S_{\infty }^{i}\left(x_{\mathrm{obj}}^{i}\right).$$

Only scattering removal is involved in this method. The attenuation was not removed in this image; thus, it still suffers from poor contrast. From that physical meaning, we call ${\hat{R}}_{n}^{i}\left(x_{\mathrm{obj}}^{i}\right)$ a descattered image. However, we will show in Section 4 that this method is feasible for stereo vision in uniform steam environments. However, it fails in the case of non-uniform steam. 

**Figure 4:**
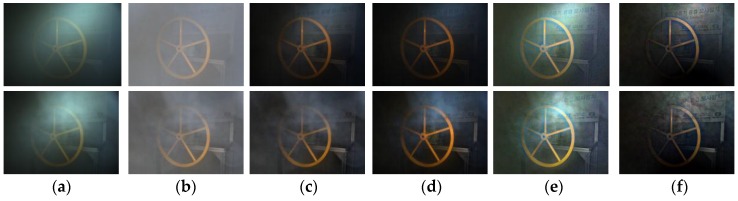
Descattering and defogging result. (**a**) Corrupted images; (**b**) Light compensated image; (**c**) Our descattered result; (**d**) Our defogged result; (**e**) Nighttime dehazing from Zhang et al. [35]; (**f**) Nighttime dehazing from Li et al. [36].

Figure 4 shows our descattered and defogged results. The first row shows images when the fog is uniform while the second row depicts the images in the case of non-uniform fog. The image in Figure 4a was taken in a very dense fog environment $\left(c=1.6\;m^{-1}\right)$ associated with lighting setup 2 in Figure 3. In Figure 4b, the light compensated image ${\hat{J}}^{i}\left(x_{\mathrm{obj}}^{i}\right)$ of the input image is illustrated, which were scaled into [0,1] for visualization. Figure 4c,d show descattered and defogged results from the proposed method, respectively. Figure 4e,f are nighttime dehazed results of Zhang et al. [35] and Li et al. [36], respectively. In the case of uniform fog, it can be seen that both descattered and defogged images from our method are better than that of [35,36].

The method of [35] is incapable of removing image glow whereas in the result of [36], the dark area becomes very dark. In the case of non-uniform fog, our defogged method and the method in [36] show better ability of non-uniform fog removal. The result of [36], however, still makes the dark area become darker.

## 4. Stereo Vision Results

### 4.1. Experimental Setup

- In the first setting, the stereo baseline is 10 cm. The light is put under the cameras. The light source and cameras are not coaxial. The experiment was conducted in a booth with dimensions of 3 × 1.5 × 1.6 m^3^. We utilized a steam generator to generate the steam using pure water inside the cabin. The generated steam’s temperature is 100–120 °C. Our system is able to produce steam as dense as an attenuation coefficient of 1.15 m^−1^.
- In the second setup, the stereo vision is the same as the previous configuration. However, the light source is placed above the cameras and coaxial to cameras. This experiment was done in a room with dimensions of 6 × 4 × 2.5 m^3^. To generate fog in such a big room, we utilized a fog machine (CHAMP-1500W, Joongang Special Lights, Seoul, Korea) that uses oil.

We make use of visibility to estimate the steam and fog density. The visibility is a measure of distance at which an object can be clearly discerned from the background. Visibility V is calculated as:

(26) $$V=\frac{C}{c}$$

where C is a constant depending on contrast ratios. Contrast ratios are between 0.018 and 0.03. A contrast ratio of 0.02 is usually used to calculate the visual range; thus, C=3.912. The attenuation coefficient is calculated as follows:

(27) $$c=\frac{1}{L}\ln\frac{I}{I_{0}}$$

where *L* is the distance that the light travels from the source to the receiver. $I_{0}$ and I are the intensity measured when light travels in the clear condition and the foggy condition, respectively. To measure the attenuation coefficient c and then visibility V, a HeNe laser (wavelength of 632.8 nm and power of 0.8 mW) and a photodiode sensor (S120C), both from Thorlabs, Newton, NJ, USA, were employed as an emitter and receiver, respectively. It should be noted that the attenuation coefficient c is wavelength-dependent. The longer the wavelength is, the higher the attenuated coefficient c is.

### 4.2. Stereo Results from Synthetic Images

Twelve datasets (Middlebury 2014 stereo datasets) from [9] were selected and used to generate synthetic data. The images were resized by half. We created synthetic images based on our imaging model derived in Section 2 with the provided ground truth disparity map. We normalized and scaled the ground truth depth map into a range from 0.5 m to 2.5 m. In the attenuated signal term, the non-uniformity of the illumination source is negligible. Only the attenuation of object radiance (from the original images) is considered. A backscattering signal is added to images based on our real pre-calibrated saturated backscattering signal $S_{\infty }^{i}\left(x_{\mathrm{obj}}^{i}\right)$.

The criteria to evaluate the quality of the disparity map from the synthetic image is the percentage of good matching pixels [8]. The threshold value of one was used. If the difference between the estimated disparity and the ground truth is larger than one, the pixel is considered to be a bad pixel. Otherwise, it is a good pixel.

We found that our descattered images ${\hat{R}}_{n}^{i}\left(x_{\mathrm{obj}}^{i}\right)$, derived in Section 3.2.2, without DCP-based defogging provide a better stereo result in the case of dense uniform steam. However, for images of non-uniform steam scenes, the defogged images ${\hat{L}}_{\mathrm{obj}}^{i}\left(x_{\mathrm{obj}}^{i}\right)$, derived in Section 3.2.1, work better. The reason is that the defogging algorithm based on DCP is based on statistics; thus, the estimation of transmission may not be accurate. Therefore, the color, which is very sensitive to the transmission map, in left and right images is less similar after defogging, which causes wrong matching. The descattered image, on the other hand, is very close to the modified object radiance. The reason is that the modified transmission $\tau^{k}\left(x_{\mathrm{obj}}\right)$ is almost constant and close to 1 in a dense scatterer environment. In the case of non-uniform fog or steam, because *c* and *k* are spatial-varying, the above assumption does not hold. In this case, DCP based defogging can remove the non-uniformity of the fog in the image; thus, the stereo vision quality of defogged images is better than that of descattered images. This will be proven in both synthetic images in this section and real images in the next section.

Semi-global matching (SGM) [44] was employed as a stereo vision algorithm in our real robot manipulation task. Table 1 shows a comparison of the disparity map quality between the descattered and defogged images of two kinds of conditions, namely, uniform steam (*V* = 3 m) and non-uniform steam (V∈[3,4] m). When dealing with images corrupted by uniform dense steam, descattered images are about 10% better than defogged images. In the case of non-uniform steam, defogged images, however, provide a 7% better result. Thus, the choice of making use of descattered images or defogged images depends on whether the environment is uniform.

For evaluation, we compared the disparity map from our descattering and defogging method with those of backscatter-corrupted images, Negahdaripour and Sarafraz [40], Zhang et al. [35], and Li et al. [36]. The method in [40] improves stereo matching by incorporating backscattering cues. This method is a local matching method and can obtain the depth map directly. The authors utilized Normalized Sum of Square Difference (NSSD) with the mean subtraction function as the matching cost. The nighttime dehazing methods in [35,36] can improve the visibility of a hazed image of a scene illuminated by active light sources. We implemented the method in [40] and ours using Matlab, while the authors of [35,36] provided their software run in C and Matlab, respectively. We can freely choose the stereo algorithm to process our descattered and defogged images. However, since the method in [40] is based on NSSD, we treat the other images in the stereo vision step using the same matching cost function for a fair comparison. It should be noted that in our robot manipulation, we employed SGM.

**Figure 5:**
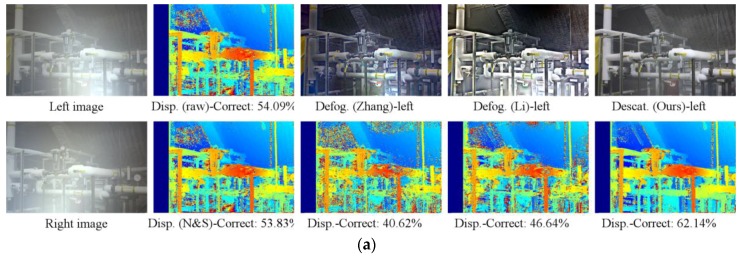

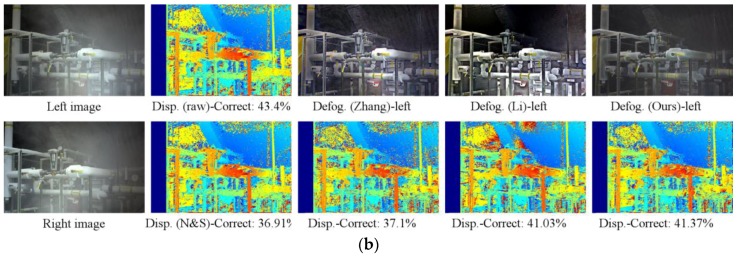
An example of synthetic images of Pipes [9]; the stereo method is NSSD: (**a**) Lighting 1—uniform; (**b**) Lighting 1—non-uniform. The first column is corrupted images. The second column shows the disparity map from input images and the one obtained by using the method in [40]. “N&S” stands for Negahdaripour and Sarafraz [40]. The third to last columns are the defogged (or descattered) images and disparity maps using the methods from [35,36] and the proposed method, respectively. “Disp.” and “Defog.” stand for disparity map and defogged image, respectivley.

**Figure 6:**
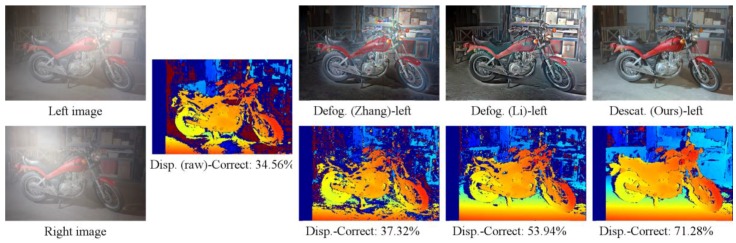
An example of synthetic images of Motor in the case of lighting 2 and uniform fog: The first column is corrupted images; the second is disparity from input images; the third to the last columns are the defogged (or descattered) images and disparity maps obatined by using the methods in [35,36] and the proposed method, respectively.

Table 2 illustrates the summarized comparison of the stereo vision results using NSSD in three conditions, namely, lighting setups 1 and 2 with uniform fog, and lighting setup 1 with non-uniform fog. The data are the average correct rate of the 12 datasets. In the case of uniform fog, our descattered images were used for stereo vision. In lighting 1, the proposed method shows at least a 14% higher correct rate than all the other methods. The stereo results obtained from corrupted images, dehazed images using the method in [36], and the stereo results obtained by using method in [40] are almost identical while the stereo results obtained from dehazed images using the method in [35] are worse than using corrupted images. There are several reasons for this. First, NSSD is capable of compensating offset and gain [45]; thus, it already works well in the case of corrupted images. As mentioned in Section 1, the method in [40] assumed that the depth in the supported window is constant, which led to wrong estimation of the scattering signal at depth discontinuities, especially in a highly non-homogeneous scattering signal area. In the datasets with lighting 1, there is strong backscattering at the high depth discontinuities areas of the datasets, as in the example of the Pipes dataset shown in Figure 5. Therefore, there is no improvement compared with the corrupted images. The method in [35] provide the worst results because this method is unable to remove the strong backscatter in the image due to their imaging model. The method in [36] has the ability to remove glow, and hence works better than that in [35]. In lighting 2, as shown in Figure 6, the light illuminates the scene above the camera; thus, a strong backscattering signal projects into the higher area of images. In these datasets, these regions have fewer depth discontinuities. Consequently, the disparity map correct rate obtained by using the method in [40] is about 11% greater than that of the original corrupted images. The nighttime dehazing methods in [35,36], and our method show the identical correct rate compared with the rate in the previous case. It should be noted that our disparity map quality is the best and is 20% higher than the disparity obtained from the input images. In the case of non-uniform steam, the results of dehazed images from [36] and our defogged images have almost the same quality and slightly higher quality than the others.

Since in the real system we employ SGM, the proposed method is also compared with backscatter-corrupted images [35,36], using SGM as the stereo algorithm, as shown in Table 3 and an example in Figure 6. In this case, SGM performs worse than NSSD when using corrupted images while it performs better using dehazed images from [35,36], and ours. When using SGM, the method in [35] provides slightly better quality than the original images. In the case of uniform fog, the proposed method improves the matching rate by about 35% and 20% compared with input images and dehazed image abtained by using the method in [36], respectively. In the case of non-uniform steam, our method and the method in [36] are nearly the same, being 10% greater than the inputs. 

### 4.3. Stereo Vision Results from Real Images

In Section 2, it is assumed that the input image $I^{i}\left(x_{\mathrm{obj}}^{i}\right)$ is given in the actual scene radiance values. The radiance maps can be recovered by inverting the acquisition response curve proposed by Debevec and Malik [42]. This is the only preprocessing step, which is employed in our experiment. This step also helps reducing variations in color which are produced by two different cameras in the stereo vision system.

**Figure 7:**
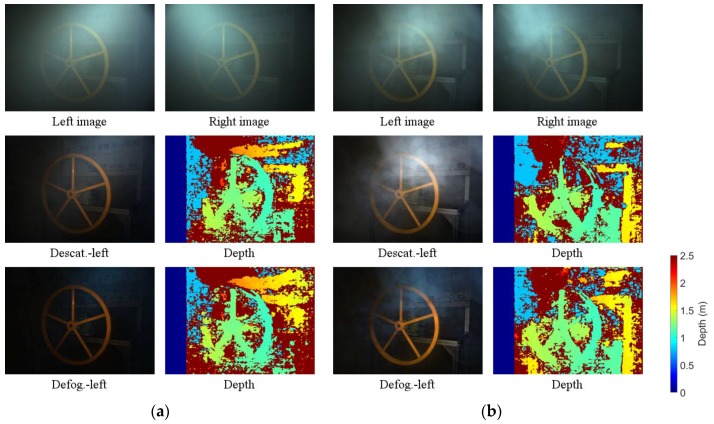
Experimental results; the stereo method is SGM: (**a**) Lighting 2—uniform fog of *V* = 2.4 m; (**b**) Lighting 2—non-uniform fog. The first row is corrupted images; the second and third row are descattered and defogged images, respectively.

Figure 7 shows a comparison of the depth map quality between the descattered and defogged images from the proposed method of two kinds of conditions, namely, uniform (*V* = 2.4 m) and non-uniform steam. When dealing with images corrupted by uniform dense steam, descattered images are better than defogged images. In the case of non-uniform steam, defogged images, however, provide better result. This is consistent with the simulation results as shown in Table 1.

**Figure 8:**
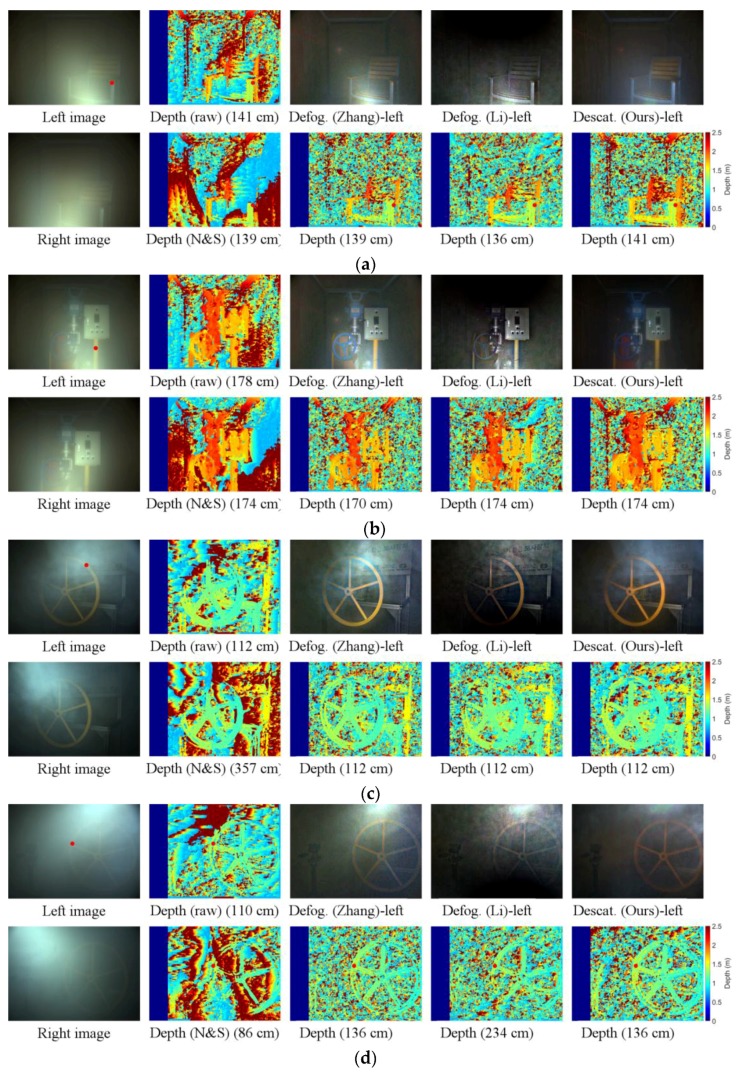
Experimental results; the stereo method is NSSD: (**a**) Lighting 1—*V* = 4.24 m; (**b**) Lighting 1—*V* = 3.39 m; (**c**,**d**) Lighting 2—non-uniform. The first column is corrupted images; the second column shows the depth maps from input images and those obtained by using method in [40]; the third to last columns are the defogged (or descattered) images and disparity maps using methods in [35,36] and the proposed method, respectively. The number under the every depth map is the measured depth at the red dot.

**Figure 9:**
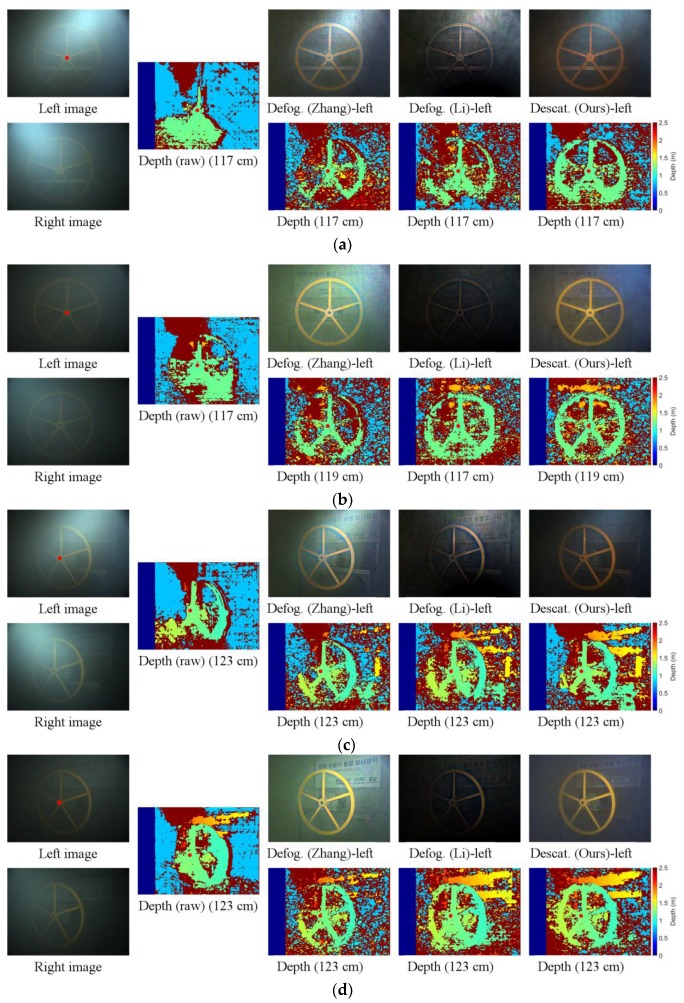
Experimental results; the stereo method is SGM: (**a**) Lighting 2—*V* = 1.71 m and polarization angle of 45°; (**b**) Same as (**a**) with polarization angle of 90°; (**c**) Lighting 2—*V* = 2.39m and polarization angle of 45°; (**d**) Same as (**c**) with polarization angle of 90°;. The first column is corrupted images; the second is disparity from input images; the third to last columns are the defogged (or descattered) images and disparity maps obatined using methods in [35,36] and the proposed method, respectively. The number under the every depth map is the measured depth at the red dot.

We depicted several real experiment data in Figure 8 and Figure 9. Figure 8a,b show two examples of lighting setup 1 in dense uniform steam (*V* are 4.24 and 3.39 m) using NSSD. In Figure 8a, the proposed method performs the best and more depth detail can be reconstructed while [40] shows the worst result in reconstructing the chair. The reason for this is the assumption of [40] as mentioned in the previous section. The method in [40], however, has better ability to estimate the background depth. Figure 8b shows a similar trend. Figure 8c,d illustrate examples of non-uniform fog under setup 2 using NSSD. In both cases, the valve is tilted at an angle of 20° to 30° compared with cameras’ optical axis and the distance from the center of the valve to cameras is 1.2 m. In both cases, the proposed method outperforms the input images, [35,36,40], in constructing the depth of object, especially the valve. The depth results from input images and that obtained by using method in [40] are the worst in both cases, especially in strong backscattering regions. In Figure 8d, the method in [35] performs better than that in [36] because the dehazed images of [36] are very dark in the lower areas.

Figure 9 depicts examples under setup 2 using SGM and the effect of polarization. In Appendix A, we discuss about the active polarization lighting and the effects of polarization. Figure 9a,c show two examples of lighting setup 2 in dense uniform steam (V are 1.71 and 2.39 m) when the polarization angle is 45°. In both cases, the distance from the center of the valve to cameras is 1.2 m. Figure 9b,d show data under the same conditions as Figure 9a,c, respectively, when the polarization angles are 90°. In both cases, the proposed method outperforms the input images [35,36], in reconstructing the depth of the object, especially the valve.

For every method, utilizing orthogonal polarization provides a better result than using a polarization angle of 45°. Directly using input images does not work well in both polarization angles. One important observation is that all methods can estimate the distance to the center of the valve accurately. Our system is better since it provides more constructed points. Finally, another crucial factor to utilize the vision algorithm in a real robot application is real-time capability. Table 4 shows the processing time to obtain the descattered or defogged images. We took the average processing time when processing 100 images continuously. The software and code run in different environments. Authors of [35] provided their software, which is an executable file in C++ environment, while authors of [36] provided a protected function run in Matlab. We implemented our descattering and defogging method using Matlab (non-optimized implementation). Thus, this is not a fair comparison. Nevertheless, we demonstrate a near real-time capability of our descattering method to enhance the input images for the stereo vision system with a processing time of 34 ms for a single image. 

## 5. Verification with Robot Manipulation

To verify the proposed algorithm, we successfully demonstrated robot manipulation in a foggy condition. In this chapter, the robot system of the manipulator is introduced, and the results of a valve turning mission in a foggy condition are presented. 

### 5.1. The Robot System of the Manipulator

The robot manipulator is constructed with seven actuators (shoulder: three axes, elbow: one axis, and wrist: three axes) to mimic the human arm configuration, which is a redundant system. The actuator models used in the robot manipulator are PRL+120, ERB-145, and ERB-115, which are produced by SCHUNK Corporation (Mengen, Germany). The specifications of the actuator model are given in Table 5.

### 5.2. Manipulation Experiment in Foggy Condition

We performed a manipulation experiment in foggy conditions to verify the effectiveness of the descattering method in a real robotics application.

#### 5.2.1. Experiment Environment

**Figure 10:**
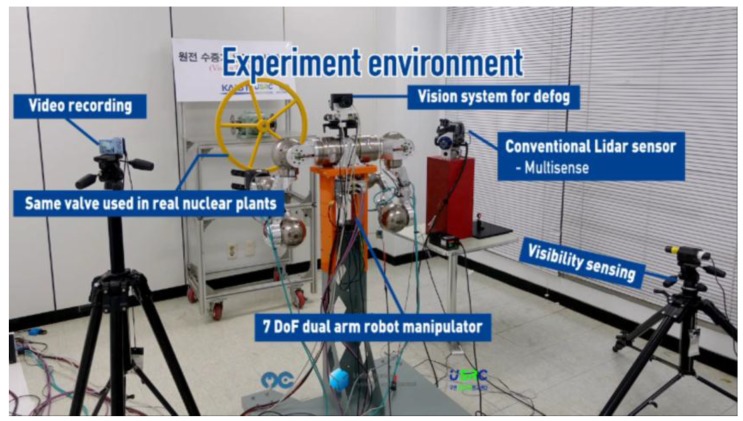
Experiment environment of valve turning manipulation in foggy condition.

The experiment environment is illustrated in Figure 10. The LiDAR (MultiSense SL from Carnegie Robotics, Pittsburgh, PA, USA) is also placed in the experiment environment for comparison. With the laser-based visibility measurement system, we monitor the visibility. To generate the fog, we used the fog machine, which has a power of 1500 W. 

**Figure 11:**
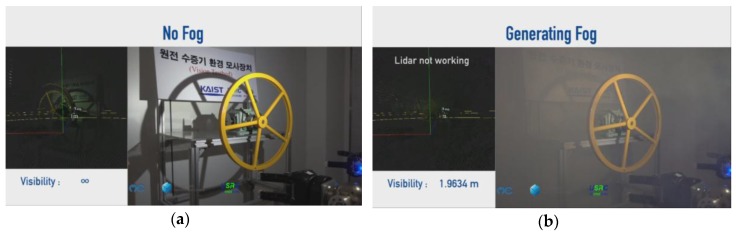
Visibility comparison: (**a**) without fog and (**b**) dense fog.

With the fog machine, the foggy condition where the visibility range is under 2 m can be generated in experimental setup 2, as explained in the previous section. As seen in Figure 11, the LiDAR works well in a clear environment. However, in the dense fog condition, it is unable to work.

#### 5.2.2. Experiment Results

**Figure 12:**
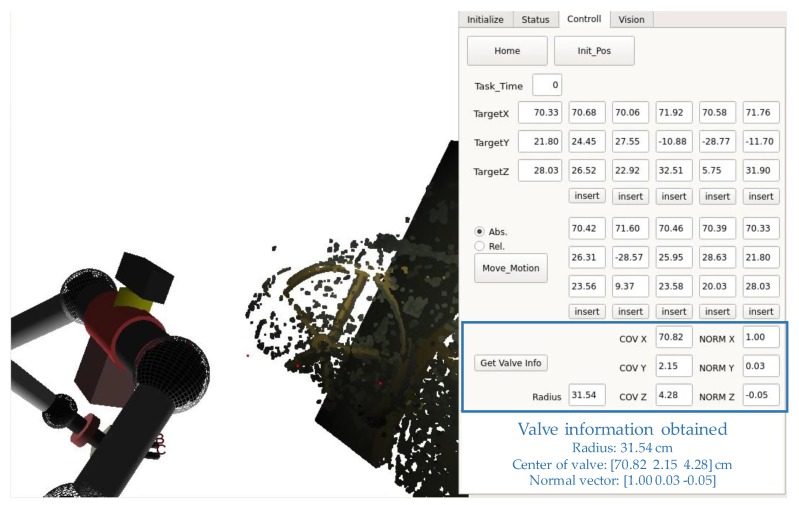
The obtained valve information.

With the proposed descattering-then-stereo algorithm, we are able to obtain a depth map. Based on the depth map, points of the valve are manually selected by the user. From these points (for example, 10 points), the center coordinate, normal vector, and radius of the valve is accurately extracted in a foggy condition. As shown in Figure 12, the obtained radius, the position of the center, and the normal vector of the valve are 31.54 cm, (70.82, 2.15, 4.28 cm), and (1.00, 0.03, −0.05), respectively.

**Figure 13:**
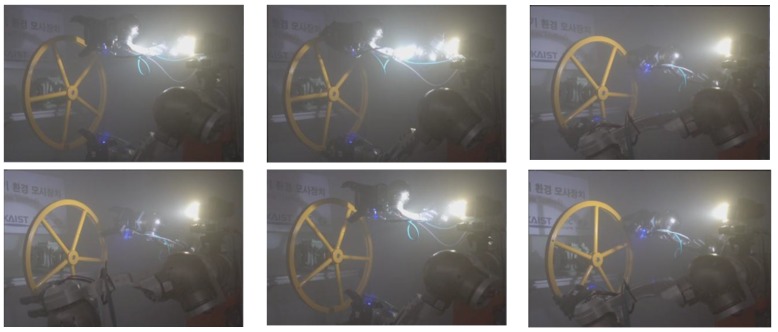
The snapshot of the robot turning the valve in dense fog condition.

With the valve information, the mission to turn the valve is successfully performed, as shown in Figure 13. The operator controls the robot remotely only using the vision data. As shown in Figure 9, backscatter-corrupted images generate poor quality depth maps. Therefore, although our method does not directly benefit the manipulation task, it helps providing higher quality input images for stereo vision. More specifically, our method reconstructs denser depth maps, from which we can select more points from a larger variety of positions to produce a more accurate estimation.

## 6. Conclusions

In this paper, we present our descattering method, which can enhance images corrupted by strong non-uniform backscattering from an active illumination source. The method is very promising since it can enhance images for stereo vision and it is near real-time capable.

It is worth noting that our method is a model-based method. The proposed method and method from [40] are based on the pre-calibrated saturated backscattering. Thus, it is not surprising that our method outperforms the methods from [35,36]. However, we have proposed a simple method that is able to enhance the images of dense fog or dense steam scenes very efficiently for stereo vision. The method is not restricted to our application. It can be utilized in other applications where active lighting is necessary, such as underwater robots.

An important issue in using our method is the choice whether to use descattered images or defogged images, such that a uniform fog/steam environment requires descattered images while a non-uniform environment requires defogged images. In practical operation, as mentioned in Section 5, the operator controls the robot remotely using vision data. The operator is also the one to make this decision. Algorithm to automatically detect non-uniform (heterogeneous) fog environment would be an issue for future works.

## Figures and Tables

**Table 1:** Correct matching rate from proposed descattered and defogged images.

Dataset Name	Uniform Fog		Non-Uniform Fog	
	Descat (%)	Defog (%)	Descat (%)	Defog (%)
Adirondack	67.10	52.79	30.84	42.73
Backpack	76.69	72.13	43.84	51.1
Cable	61.72	40.37	15.90	19.33
Classroom1	85.87	64.49	10.04	24.79
Flowers	44.82	48.63	19.04	14.56
Motorcycle	76.22	71.86	43.32	58.35
Pipes	66.96	58.78	49.19	49.68
Recycle	67.23	53.2	15.59	25.43
Shelves	47.45	40.44	24.88	35.63
Storage	61.77	54.75	33.81	33.21
Sword1	77.84	68.87	39.73	50.75
Sword2	42.21	27.99	6.85	11.82
**Average**	**64.66**	**54.53**	**27.75**	**34.78**

**Table 2:** Evalution of stereo vision from scatter-corrupted images; the stereo vision method is NSSD.

Lighting	Corrupted Image (%)	[40] (%)	[35] (%)	[36] (%)	Proposed Method (%)
Setup 1—uniform	33.12	33.03	25.12	34.30	47.84
Setup 2—uniform	26.39	37.29	23.04	32.82	46.16
Setup 1—non-uniform	23.70	19.37	22.25	25.70	25.99

**Table 3:** Evalution of stereo vision from scatter-corrupted images; the stereo vision method is SGM.

Lighting	Corrupted Image (%)	[35] (%)	[36] (%)	Proposed Method (%)
Setup 1—uniform	28.09	31.31	45.46	64.66
Setup 2—uniform	19.49	26.99	41.89	55.53
Setup 1—non-uniform	24.94	29.14	34.57	34.78

**Table 4:** Processing time.

Resolution	Zhang et al. [35] (ms)	Li et al. [36] (ms)	Ours (ms)	
			Descat	Defog
780 × 580	17,470	20,520	34	860

**Table 5:** Specifications of the actuators.

Specification	Uint	ERB-115	ERB-145	PRL-120
Max Speed	°/s	72	72	25
Nominal Torque	Nm	7	35	216
Max Torque	Nm	19	64	372
Max rotation angle	°	340	340	360
Weight	kg	1.8	3.9	3.6

**Figure A1:**
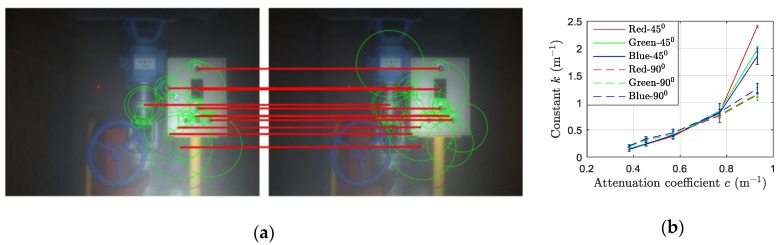
Effect of polarizization: (**a**) Correspondences between left-right images; (**b**) Constant *k* estimation, which is attenuation coefficient dependent.

**Figure A2:**
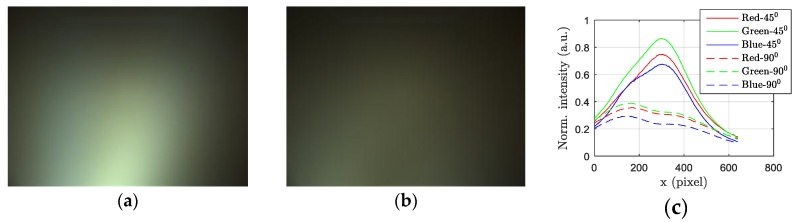
Effect of polarization in saturated backscattering: (**a**) Saturated backscattering of left camera when the polarization angle is 45°; (**b**) Saturated backscattering of left camera when the angle of polarization is 90°; (**c**) Intensity along x-axis at the section where intensity attains the highest value.

## Appendix A. Polarization-Based Backscattering Removal

Using artificial light that is close to the camera, as in our system, causes a strong backscatter. To partly remove the strong backscattering, we utilize polarization, which has proven excellent ability to reduce backscattering [23,24,46,47,48]. We simply modify the conventional image acquisition system to make active polarization, similar to [23,24], by adding three polarizers: each one is mounted in front of the light source and every camera in the stereo vision system. To ensure photo-consistency, we aligned the polarizers in front of the two cameras so that they are in the same state. We use polarization angle to call the angle between the state of the polarizer of the light source and the state of the polarizers of the two cameras. This system provides a pure optics-based scattering removal for improving visibility. The best situation for backscattering removal is when the polarization angle is 90° [23,24]. Through our experiment, we found that it is very feasible to utilize the active polarizer. The active polarization lighting reduces both saturated backscattering and the constant parameter *k*.

To estimate the parameter *k,* we first apply the initial step of our proposed descattering method, as in Equation (16). The feature extraction and matching are then done by employing speeded up robust features (SURF) [49]. It should be noticed that SURF is unable to detect any feature when applied to the original corrupted images. The constant *k* can be estimated as:

(A1) $$k=\frac{\sum_{j=1}^{N}k_{j}}{N}$$

where *N* is the number of detected features; *j* is the feature index; and $k_{j}$ can be easily obtained by inverting Equation (13), noting that the attenuated radiance in the left and right images are the same:

(A2) $$k_{j}=-\frac{1}{\left\|X_{\mathrm{obj},j}^{L}\right\|}\ln\left[1-\frac{I^{L}\left(x_{\mathrm{obj},j}^{L}\right)-I^{R}\left(x_{\mathrm{obj},j}^{R}\right)}{S_{\infty }^{L}\left(x_{\mathrm{obj},j}^{L}\right)-S_{\infty }^{R}\left(x_{\mathrm{obj},j}^{R}\right)}\right].$$

Figure A1 depicts an estimation of the constant *k* and the effect of the active polarization on *k*. Figure A1a shows an example of feature matching between left and right images using SURF. From these matched features, *k* can be estimated by using Equations (A1) and (A2). The method was applied to images of different attenuation coefficient *c* and two different polarization angles, namely, 45° and 90°. To change states of the polarization, we fix the polarizers of the two cameras and rotate the polarizer in front of the light source. Figure A1b illustrates the relationship of *k* and *c* of two different states of polarization. Solid lines show the value of *k* when the polarization angle is 45° and dash lines depict the value when the states of polarizers of the light and the cameras are orthogonal. The figure shows that when *c* is smaller than 0.8 m^−1^, *k* and *c* are close each other. Additionally, *k* of values of different polarization angles are almost identical. When *c* is beyond 0.8, *k* in the solid lines increase dramatically with *c*. The dash lines, however, almost have a linear relationship with *c*. We could not measure *k* in denser conditions since very few features can be detected and matched. However, it can be proven that the orthogonal setup of polarization between the illuminator and receivers provides an advantage in scattering reduction because the rate at which $S\left(x_{\mathrm{obj}}^{i}\right)$ increases with $\left\|X_{\mathrm{obj}}^{i}\right\|$ set by parameter *k*. It is worth noting that *k* also depends on the lighting geometry; thus, the values shown in Figure A1 are only correct in the specific lighting geometry in our experiment. The method to measure *c* is be explained in Section 4.1.

Figure A2 shows another advantage of using polarization. Besides reducing *k*, the orthogonal setup can reduce as much as 50% of saturated backscattering of the 45° setup where the signal attains the highest value (Figure A2c). From these two figures above, it is proven that, with slight modification of the system, we can significantly remove the scattering signal. It is also well known that the specular reflection often leads to problems in stereo matching. Therefore, by removing specular reflection, we can also improve stereo matching.
